# Isatuximab plus atezolizumab in patients with advanced solid tumors: results from a phase I/II, open-label, multicenter study

**DOI:** 10.1016/j.esmoop.2022.100562

**Published:** 2022-08-18

**Authors:** M. Simonelli, E. Garralda, F. Eskens, M. Gil-Martin, C.-J. Yen, R. Obermannova, Y. Chao, S. Lonardi, B. Melichar, V. Moreno, M.-L. Yu, A. Bongiovanni, E. Calvo, S. Rottey, J.-P. Machiels, A. Gonzalez-Martin, L. Paz-Ares, C.-L. Chang, W. Mason, C.-C. Lin, D.A. Reardon, M. Vieito, A. Santoro, R. Meng, G. Abbadessa, F. Menas, H. Lee, Q. Liu, C. Combeau, N. Ternes, S. Ziti-Ljajic, C. Massard

**Affiliations:** 1 IRCCS Humanitas Research Hospital, Rozzano, Italy; 2 Department of Biomedical Sciences, Humanitas University, Pieve Emanuele, Italy; 3 Vall d’Hebron Institute of Oncology, Barcelona, Spain; 4 Erasmus MC Cancer Institute, Rotterdam, the Netherlands; 5 Institut Català d’Oncologia-IDIBELL, L’Hospitalet, Barcelona, Spain; 6 National Cheng Kung University, Tainan, Taiwan; 7 Masaryk Memorial Cancer Institute, Brno, Czech Republic; 8 Department of Oncology, Taipei Veterans General Hospital, Taipei, Taiwan; 9 Veneto Institute of Oncology IOV, IRCCS, Padova, Italy; 10 Department of Oncology, Palacky University, Olomouc, Czech Republic; 11 START Madrid-FJD, Hospital Fundación Jiménez Díaz, Madrid, Spain; 12 Hepatobiliary Division, Department of Internal Medicine and Hepatitis Center, Kaohsiung Medical University Hospital, Kaohsiung Medical University, Kaohsiung, Taiwan; 13 IRCCS Istituto Romagnolo per lo Studio dei Tumori (IRST) “Dino Amadori”, Meldola, Italy; 14 START Madrid-CIOCC, Centro Integral Oncológico Clara Campal, Madrid, Spain; 15 Ghent University, Ghent; 16 Cliniques Universitaires Saint-Luc, Brussels, Belgium; 17 Clínica Universidad de Navarra, Madrid, and Program in Solid Tumors, Center for Applied Medical Research (CIMA), Pamplona; 18 Hospital Universitario 12 de Octubre, Madrid, Spain; 19 Department of Obstetrics and Gynecology, Mackay Memorial Hospital, Taipei, Taiwan; 20 Princess Margaret Cancer Centre, Toronto, Canada; 21 National Taiwan University Hospital, Taipei, Taiwan; 22 Dana-Farber Cancer Institute, Harvard University, Boston; 23 Sanofi, Cambridge, USA; 24 Sanofi, Chilly-Mazarin, France; 25 Gustave Roussy, Villejuif, France

**Keywords:** isatuximab, atezolizumab, anti-CD38, anti-PD-L1, solid tumors

## Abstract

**Background:**

The anti-CD38 antibody isatuximab is approved for the treatment of relapsed/refractory multiple myeloma, but there are no data on its efficacy in solid tumors. This phase I/II study (NCT03637764) assessed the safety and activity of isatuximab plus atezolizumab (Isa + Atezo), an anti-programmed death-ligand 1 (PD-L1) antibody, in patients with immunotherapy-naive solid tumors: epithelial ovarian cancer (EOC), glioblastoma (GBM), hepatocellular carcinoma (HCC), and squamous cell carcinoma of the head and neck (SCCHN).

**Patients and methods:**

Phase I assessed safety, tolerability, pharmacokinetics, pharmacodynamics, and the recommended phase II dose (RP2D) of isatuximab 10 mg/kg intravenously (i.v.) every week for 3 weeks followed by once every 3 weeks + atezolizumab 1200 mg i.v. every 3 weeks. Phase II used a Simon’s two-stage design to assess the overall response rate or progression-free survival rate at 6 months (GBM cohort). Interim analysis was carried out at 6 months following first dose of the last enrolled patient in each cohort. Pharmacodynamic biomarkers were tested for CD38, PD-L1, tumor-infiltrating immune cells, and FOXP3+ regulatory T cells (Tregs) in the tumor microenvironment (TME).

**Results:**

Overall, 107 patients were treated (EOC, *n* = 18; GBM, *n* = 33; HCC, *n* = 27; SCCHN, *n* = 29). In phase I, Isa + Atezo showed an acceptable safety profile, no dose-limiting toxicities were observed, and RP2D was confirmed. Most patients experienced ≥1 treatment-emergent adverse event (TEAE), with ≤48.5% being grade ≥3. The most frequent TEAE was infusion reactions. The study did not continue to stage 2 based on prespecified targets. Tumor-infiltrating CD38+ immune cells were reduced and almost cleared after treatment. Isa + Atezo did not significantly modulate Tregs or PD-L1 expression in the TME.

**Conclusions:**

Isa + Atezo had acceptable safety and tolerability. Clinical pharmacodynamic evaluation revealed efficient target engagement of isatuximab via treatment-mediated reduction of CD38+ immune cells in the TME. Based on clinical data, CD38 inhibition does not improve responsiveness to PD-L1 blockade in these patients.

## Highlights

• The combination of Isa + Atezo was well tolerated, showing a manageable safety profile.
• Tumor-infiltrating CD38+ immune cells were reduced and almost cleared after treatment.
• Isa + Atezo did not significantly modulate Tregs or PD-L1 expression in the TME.
• CD38 inhibition does not improve responsiveness to PD-L1 blockade in these patients.

## Introduction

Monoclonal antibodies targeting programmed cell death protein 1/programmed death-ligand 1 (PD-1/PD-L1) alone or in combination with other anticancer agents have revolutionized the treatment landscape of different malignancies, with approvals granted in multiple indications. However, despite the success of these agents, only a minority of patients derive long-term benefits, and extensive efforts are ongoing to understand the underlying mechanisms of primary and acquired resistance.

CD38 is a multifunctional molecule member of the ribosyl cyclase family that is widely expressed on the surface of multiple immune cell types, with a key role in regulating lymphocyte development, activation, and differentiation.^1^ Overexpression of CD38 has been well documented not only in several hematological malignancies, including multiple myeloma (MM), lymphomas, and leukemias, but also in some solid tumors, such as prostate cancer and glioblastoma (GBM), while in other cancer types it can be found within the tumor microenvironment (TME). Although its activity has not been fully elucidated yet, several findings indicate that the CD38 pathway may significantly contribute to the immune-suppressive TME. Tumor up-regulation of CD38 induced by all-*trans*-retinoic acid and interferon-β has been implicated in T-cell exhaustion and may represent a major mechanism of acquired resistance to immune checkpoint blockade therapy.^2^^,^^3^ The combination of PD-L1 and CD38 blockade led to enhanced antitumor activity, decreased incidence of lung metastasis, and increased tumor infiltration of CD8+ T cells in a murine lung cancer model (K-ras^LA1/+^ p53^R172HΔg/+^-derived tumor). Based on these data, there is a strong rationale for clinically testing the combination of anti-CD38 blockade with anti-PD-1/PD-L1 agents to prevent or overcome treatment resistance and further enhance antitumor efficacy.

Based on the phase III ICARIA-MM and IKEMA studies, isatuximab (Sarclisa), a monoclonal antibody that targets a specific epitope of CD38, is approved in a number of countries in combination with pomalidomide and dexamethasone or carfilzomib and dexamethasone, for the treatment of adult patients with relapsed/refractory MM who have received prior therapy.^4^, ^5^, ^6^ Currently, there are no data on the activity of isatuximab in solid tumors. Atezolizumab (Tecentriq) is an anti-PD-L1 antibody approved for the treatment of patients with locally advanced or metastatic urothelial carcinoma, metastatic non-small-cell lung cancer [NSCLC; as adjuvant treatment; in combination with bevacizumab, paclitaxel, and carboplatin (no *EGFR* or *ALK* aberrations); in combination with paclitaxel protein-bound and carboplatin (no *EGFR* or *ALK* aberrations)], small-cell lung cancer (in combination with carboplatin and etoposide), hepatocellular carcinoma (HCC; in combination with bevacizumab), and *BRAF V600* mutation-positive unresectable or metastatic melanoma (in combination with cobimetinib and vemurafenib).^7^

The present study was designed to assess the safety and antitumor activity of isatuximab plus atezolizumab (Isa + Atezo) in immunotherapy-naive patients with advanced solid tumors who received at least one previous line of treatment for their advanced disease. Types of tumors included platinum-resistant/refractory epithelial ovarian cancer (EOC), recurrent GBM, unresectable HCC, and platinum-refractory recurrent squamous cell carcinoma of the head and neck (SCCHN). Cohort selection for this trial was based on immune responsiveness, with HCC and SCCHN being moderately immune-responsive to anti-PD-1/PD-L1 monotherapy, and GBM and EOC being immune-resistant cancers for which PD-1/PD-L1 axis blockade alone has not been shown to be effective.

### Patients and methods

#### Study design and patients

This was an open-label, multicenter, phase I/II study (NCT03637764) designed to evaluate the safety, preliminary efficacy, pharmacokinetics (PK), and pharmacodynamics of Isa + Atezo in patients with advanced solid malignancies. The study comprised two parts. Phase I, the safety run-in, characterized safety and tolerability, and assessed the recommended phase II dose (RP2D) of Isa + Atezo following a 21-day dose-limiting toxicity (DLT) observation period. Phase II used a Simon’s two-stage design in four different expansion cohorts, with overall response rate (ORR), for the EOC, HCC, and SCCHN cohorts, or progression-free survival at 6 months (PFS-6), for the GBM cohort, as the primary endpoint.

The study was conducted in accordance with consensus ethics principles derived from international ethics guidelines, including the Declaration of Helsinki, the International Conference for Harmonisation guidelines for Good Clinical Practice, all applicable laws, rules, and regulations. The protocol was approved by the ethics committees of all participating centers. Informed consent was obtained before the conduct of any study-related procedures.

### Treatment

In phase I, the starting dose was 1200 mg once every 3 weeks for atezolizumab, with isatuximab given 10 mg/kg once weekly for 3 weeks followed by every 3 weeks. In case of occurrence of DLT, a dose level minus 1 with isatuximab given at 5 mg/kg was planned, while alternative dosing schedules could have been considered based on safety.

The end of treatment occurred 30 (± 7) days after final administration of study regimen or receipt of another anticancer therapy, whichever occurred first. Treatment continued until disease progression, unacceptable toxicity, patient’s decision to stop treatment, or 2 years of uninterrupted delivery of study drugs was reached without documented progressive disease.

### Immunohistochemistry and multiplex immunofluorescence assays

Single-plex immunohistochemistry (IHC) assay used PD-L1 (Ventana, SPC263, ready to use; Roche Diagnostics, Indianapolis, IN) or CD38 antibodies (Leica, SPC32, working concentration 1 : 400; Buffalo Grove, IL) optimized for detection with the OptiView DAB IHC Detection kit on the Ventana Benchmark Ultra platform.

Patients with paired screening and on-treatment biopsies were selected for the multiplexed immunofluorescent platform (MultiOmyx; NeoGenomics, Fort Myers, FL) analysis, utilizing a pair of Cy3- or Cy5-labeled antibodies per staining round. Formalin-fixed paraffin-embedded tissues were stained with a customized panel to quantify infiltrating immune cells in the TME according to the vendor-recommended protocol (NeoGenomics).

### Study assessments

Patients who received at least one dose of Isa + Atezo were evaluated for safety, tolerability, PK, pharmacodynamics, and preliminary efficacy profiles. Safety was determined by the National Cancer Institute Common Terminology Criteria for Adverse Events v4.03. Additionally, investigators assessed whether adverse events (AEs) were treatment-related or caused by other factors. DLT criteria are listed in the Supplementary Methods and Results, available at https://doi.org/10.1016/j.esmoop.2022.100560. ORR (complete response/partial response) and PFS-6 were assessed by RECIST v1.1 for solid tumors and Response Assessment in Neuro-Oncology (RANO) criteria for GBM.

### PK analysis

Blood samples were collected at prespecified timepoints for PK evaluation of isatuximab (days 1, 4, 8, and 15) over the first cycle and then at pre-dose for subsequent cycles. Isatuximab plasma concentrations were determined using a validated immunoassay (lower limit of quantification: 5 μg/ml). Non-compartmental analysis was conducted with Phoenix WinNonLin v8.1 (Pharsight, Sunnyvale, CA).

### Statistical analyses

An interim analysis was carried out at 6 months following first treatment of the last enrolled patient in each cohort. Efficacy and safety analyses were conducted using the all-treated population. Data from each cohort in phase II were analyzed and reported separately using descriptive statistics. Continuous data were summarized using mean, standard deviation, median, minimum, and maximum. Categorical and ordinal data were summarized using number and percentage.

In phase I, the actual sample size will vary depending on DLTs observed and the number of dose levels explored. Patients who are not assessable for DLT assessment in the phase I part of the study may be replaced.

The phase II part of the study is to evaluate initial antitumor activity based on tumor response using RECIST v1.1 criteria for EOC, HCC, and SCCHN and using RANO criteria for GBM. The efficacy evaluation is based on Simon’s two-stage design with 85% power at 5% one-sided α level for each of the cohorts. The assumptions of response rate, the required sample sizes, and the number of responses at each stage are provided in the Supplementary Material, available at https://doi.org/10.1016/j.esmoop.2022.100560, for EOC, HCC, and SCCHN.

## Results

### Patients

Overall, 107 patients with advanced solid tumors were enrolled, including 18 with platinum-resistant/refractory EOC, 33 with recurrent GBM, 27 with advanced unresectable HCC, and 29 with platinum-refractory recurrent SCCHN. The median patient age was 60 (range: 21-82) years, 64.5% were men, 55.4% had an Eastern Cooperative Oncology Group performance score of 1 (EOC, HCC, and SCCHN cohorts), and 54.5% had a Karnofsky performance status of ≥90 (GBM cohort). Data for individual cohorts are shown in Table 1.

**Table 1:** Summary of demographics and other baseline characteristics: all-treated population

	EOC (*n* = 18)	GBM (*n* = 33)	HCC (*n* = 27)	SCCHN (*n* = 29)	Overall (*n* = 107)
Median age, years (range)	55.0 (35-80)	55.0 (21-75)	62.8 (42-82)	62.0 (40-76)	60.0 (21-82)
Sex, *n* (%)					
Male	0	23 (69.7)	20 (74.1)	26 (89.7)	69 (64.5)
Female	18 (100.0)	10 (30.3)	7 (25.9)	3 (10.3)	38 (35.5)
ECOG performance status, *n* (%)					
0	12 (66.7)	NA	14 (51.9)	7 (24.1)	33 (44.6)
1	6 (33.3)	NA	13 (48.1)	22 (75.9)	41 (55.4)
Karnofsky performance status, *n* (%)					
70	NA	4 (12.1)	NA	NA	NA
80	NA	11 (33.3)	NA	NA	NA
90	NA	8 (24.2)	NA	NA	NA
100	NA	10 (30.3)	NA	NA	NA

ECOG, Eastern Cooperative Oncology Group; EOC, epithelial ovarian cancer; GBM, glioblastoma; HCC, hepatocellular carcinoma; NA, not applicable; SCCHN, squamous cell carcinoma of the head and neck.

Among the 18 patients with EOC, 16 (88.9%) had serous carcinoma, 1 (5.6%) had endometrioid carcinoma, and 1 (5.6%) had clear cell adenocarcinoma; 16 (88.9%) were primary ovarian tumors and 2 (11.1%) originated from the peritoneum. Overall, 70.6% were resistant to platinum chemotherapy and 29.4% were refractory. A total of 4 (22.2%) patients had received one prior regimen and 14 (77.8%) were pre-treated with at least two prior regimens. Among the 33 patients with GBM, most (96.9%) entered the trial after progressing on standard chemoradiation (‘Stupp regimen’). All but 3 patients with GBM (90.9%) had isocitrate dehydrogenase 1/2 wild type, and 10 (30.3%) had a methylated O(6)-methylguanine-DNA methyltransferase promoter. All 27 patients with HCC had preserved liver function (Child-Pugh A) and intermediate-to-advanced disease (81.5% Barcelona clinic liver cancer stage C and 18.5% stage B). All patients had received sorafenib previously and had either progressed (81.5%) or stopped treatment for intolerance (29.6%). Extrahepatic disease was reported in 20 patients (74.1%). Overall, 16 (59.3%) patients had received one prior regimen and 11 (40.7%) had received more than one prior regimen. Of the 29 SCCHN tumors, 14 (48.3%) originated from the oral cavity, 4 (13.8%) from the pharynx, 8 (27.6%) from the larynx, and 3 (10.3%) from other locations. All patients in this cohort were platinum refractory, experiencing tumor recurrence or progressive disease within 6 months of the last platinum-based therapy. Human papillomavirus status was negative in 10 (34.5%) patients and unknown in 19 (65.5%) patients. Overall, 17 (58.6%) patients received one prior regimen, 10 (34.5%) received two prior regimens, and 2 (6.9%) received more than two prior regimens.

At the time of data cut-off (14 July 2020), 103 (96.3%) patients had discontinued experimental treatment due to disease progression (92.2%), patient withdrawal (4.9%), and AEs (2.9%). The median number of cycles started was 4 (range: 1-35), and the median duration of drug exposure was 12 weeks (range: 3-108 weeks).

For EOC, the median (range) number of cycles administered was 4 (1-12), and the median duration of exposure was 12.1 weeks. As of data cut-off (27 September 2019), 17 of 18 patients had discontinued treatment, primarily due to progressive disease (*n* = 16). For patients with GBM, the median (range) number of cycles administered was 3 (1-17), and the median duration of exposure was 9.1 weeks. As of data cut-off (27 September 2019), 31 of 33 patients had discontinued treatment, primarily due to progressive disease (*n* = 29). For patients with HCC, the median (range) number of cycles administered was 5 (1-18), and the median duration of exposure was 15.1 weeks. As of data cut-off (22 November 2019), 22 of 27 patients had discontinued treatment, primarily due to progressive disease (*n* = 20). For SCCHN, the median (range) number of cycles administered was 3 (1-26), and the median duration of exposure was 11.3 weeks. As of data cut-off (14 July 2020), 25 of 29 patients had discontinued treatment, primarily due to progressive disease (*n* = 22). Overall, at least one cycle was delayed for 2 (11.1%) EOC, 7 (21.2%) GBM, and 2 (7.4%) HCC patients, and for 1 (3.4%) SCCHN patient.

### Safety

The combination of Isa + Atezo showed acceptable safety and tolerability, with no new safety signals compared with the known profile of each agent used as monotherapy. No DLTs were observed and the RP2D was confirmed [isatuximab 10 mg/kg intravenously (i.v.) weekly for 3 weeks followed by every 3 weeks + atezolizumab 1200 mg i.v. every 3 weeks]. Overall, 106 (99%) patients experienced at least one treatment-emergent adverse event (TEAE), with ≤48.5% being grade ≥3 (Table 2). A total of 88 (82.2%) patients experienced TEAEs that were considered by investigators to be related to experimental treatment (TRAEs); most of these were mild to moderate in severity. Grade ≥3 TRAEs occurred in 10 (9.3%) patients, while 8 (7.5%) were considered serious TRAEs. The most frequent TRAEs were infusion reactions for all cohorts. Grade ≥3 TRAEs included dyspnea (SCCHN, *n* = 1), immune-mediated hepatitis (HCC, *n* = 1; GBM, *n* = 1), renal failure (HCC, *n* = 1), aspartate aminotransferase increased (HCC, *n* = 1), alanine aminotransferase increased (HCC, *n* = 1), infusion reaction (HCC, *n* = 1; GBM, *n* = 1), diastolic hypertension (EOC, *n* = 1), migraine (GBM, *n* = 1), and peripheral motor neuropathy (GBM, *n* = 1). Serious TRAEs included pulmonary embolism [EOC, *n* = 1 (isatuximab)], infusion reaction [EOC, *n* = 1 (isatuximab); GBM, *n* = 1 (isatuximab/atezolizumab); HCC, *n* = 1 (isatuximab)], pneumonitis [SCCHN, *n* = 1 (atezolizumab)], dyspnea [SCCHN, *n* = 1 (isatuximab/atezolizumab)], migraine [GBM, *n* = 1 (isatuximab/atezolizumab)], renal failure [HCC, *n* = 1 (isatuximab/atezolizumab)], and immune-mediated hepatitis [HCC, *n* = 1 (isatuximab/atezolizumab)]. TRAEs leading to definitive treatment discontinuation included grade ≥3 infusion reactions [*n* = 2 (1.9%)] and grade 5 immune-mediated hepatitis [*n* = 1 (0.9%)].

**Table 2:** Overview of TEAEs: all-treated population

*n* (%)	EOC (*n* = 18)	GBM (*n* = 33)	HCC (*n* = 27)	SCCHN (*n* = 29)
TEAEs (any grade)	18 (100)	33 (100)	26 (96.3)	29 (100)
TEAEs of grade ≥3	5 (27.8)	16 (48.5)	11 (40.7)	12 (41.4)
TEAEs of grade 5^a^	3 (16.7)	1 (3.0)	4 (14.8)	7 (24.1)
Serious TEAEs	7 (38.9)	8 (24.2)	10 (37.0)	16 (55.2)
TRAEs^b^ (any grade)	18 (100)	29 (87.9)	23 (85.2)	18 (62.1)
TRAEs of grade ≥3	1 (5.6)	4 (12.1)	4 (14.8)	1 (3.4)
Serious TRAEs	2 (11.1)	2 (6.1)	2 (7.4)	2 (6.9)
TEAEs leading to definitive study drug discontinuation	1 (5.6)	1 (3.0)	2 (7.4)	0
TEAEs leading to premature discontinuation of isatuximab	0	0	0	0
TEAEs leading to premature discontinuation of atezolizumab	0	0	0	0
AESI^c^	10 (55.6)	12 (36.4)	12 (44.4)	9 (31.0)
AESI of grade ≥3	0	2 (6.1)	2 (7.4)	1 (3.4)

AE, adverse event; AESI, adverse event of special interest; EOC, epithelial ovarian cancer; GBM, glioblastoma; HCC, hepatocellular carcinoma; IAR, infusion-associated reaction; IMP, investigational medicinal product; NIMP, non-investigational medicinal product; SCCHN, squamous cell carcinoma of the head and neck; TEAE, treatment-emergent adverse event; TRAE, TEAE considered by investigators to be related to experimental treatment.

a Grade 5 TEAEs were due to disease progression (EOC, *n* = 3; HCC, *n* = 3; SCCHN, *n* = 4) and other TEAEs [GBM: euthanasia (*n* = 1); HCC: immune-mediated hepatitis (*n* = 1); SCCHN: acute respiratory failure (*n* = 1), arterial hemorrhage (*n* = 1), tumor hemorrhage (*n* = 1)].

b Treatment-related TEAEs were TEAEs related to at least one drug of the combination.

c AESI included grade ≥2 IARs, grade ≥3 immune-related TEAEs, immune-related AEs of any grade in a patient previously treated with a phosphoinositide 3-kinase (PI3K) inhibitor (only applicable for patients who received atezolizumab), pregnancy, and symptomatic overdose with IMP/NIMP.

### Antitumor activity

Combining data from the EOC, HCC, and SCCHN cohorts (*n* = 74), the ORR according to RECIST criteria was 9.5%, including 1 (1.4%; SCCHN) complete response and 6 (8.1%; 3 SCCHN, 2 HCC, 1 EOC) partial responses, whereas 21 (28.4%) patients achieved stable disease as best response (Table 3). The median duration of response (DOR), median time to response, and median PFS were 4.57 (range: 2.1-14.29) months, 2.07 (range: 1.87-4.18) months, and 1.92 (95% confidence interval 1.81-2.04) months, respectively. Data for individual cohorts are included in Table 3. In the GBM cohort, no patients achieved a radiographic response according to RANO criteria, and the PFS-6 was 3.1%. Due to the fact that prespecified levels of activity were not reached for each cohort, the study did not continue with expansion to stage 2.

**Table 3:** Summary of response rates: all-treated population

	EOC (*n* = 18)	GBM (*n* = 33)	HCC (*n* = 27)	SCCHN (*n* = 29)	Overall^a^ (*n* = 74)
Best overall response, *n* (%)					
Complete response^b^	0	0	0	1 (3.4)	1 (1.4)
Partial response^b^	1 (5.6)	0	2 (7.4)	3 (10.3)	6 (8.1)
Stable disease	6 (36.3)	4 (12.1)	8 (29.6)	7 (24.1)	21 (28.4)
Progressive disease	10 (55.6)	26 (78.8)	14 (51.9)	11 (37.9)	35 (47.3)
Not assessable^c^	1 (5.6)	3 (9.1)	3 (11.1)	7 (24.1)	11 (14.9)
Unconfirmed complete response	1 (5.6)	—	0	0	1 (1.4)
Unconfirmed partial response	0	—	1 (3.7)	0	1 (1.4)
Overall response, *n* (%)					
Responders (complete or partial response)^b^	1 (5.6)	0	2 (7.4)	4 (13.8)	7 (9.5)
90% CI^d^	0.3-23.8	0.0-8.7	1.3-21.5	4.9-28.8	4.5-17.0
Median PFS (95% CI)	2.04 (1.91-3.94)	1.30 (1.22-1.38)	2.04 (1.84-4.01)	2.10 (1.84-1.91)	1.92 (1.81-2.04)^e^

CI, confidence interval; EOC, epithelial ovarian cancer; GBM, glioblastoma; HCC, hepatocellular carcinoma; PFS, progression-free survival; SCCHN, squamous cell carcinoma of the head and neck.

a Including EOC, HCC, and SCCHN cohorts, as per RECIST v1.1 criteria.

b Confirmation of response was required.

c Including patients with no target and non-target lesions identified at baseline and no new lesions reported in post-baseline tumor assessments, or patients with only non-target lesions identified at baseline with non-complete response/non-progressive disease reported for non-target lesion and no new lesions reported in post-baseline tumor assessments.

d Estimated using the Clopper–Pearson method.

e PFS estimate for all-treated population (*n* = 107).

### Biomarkers

**Figure 1:**
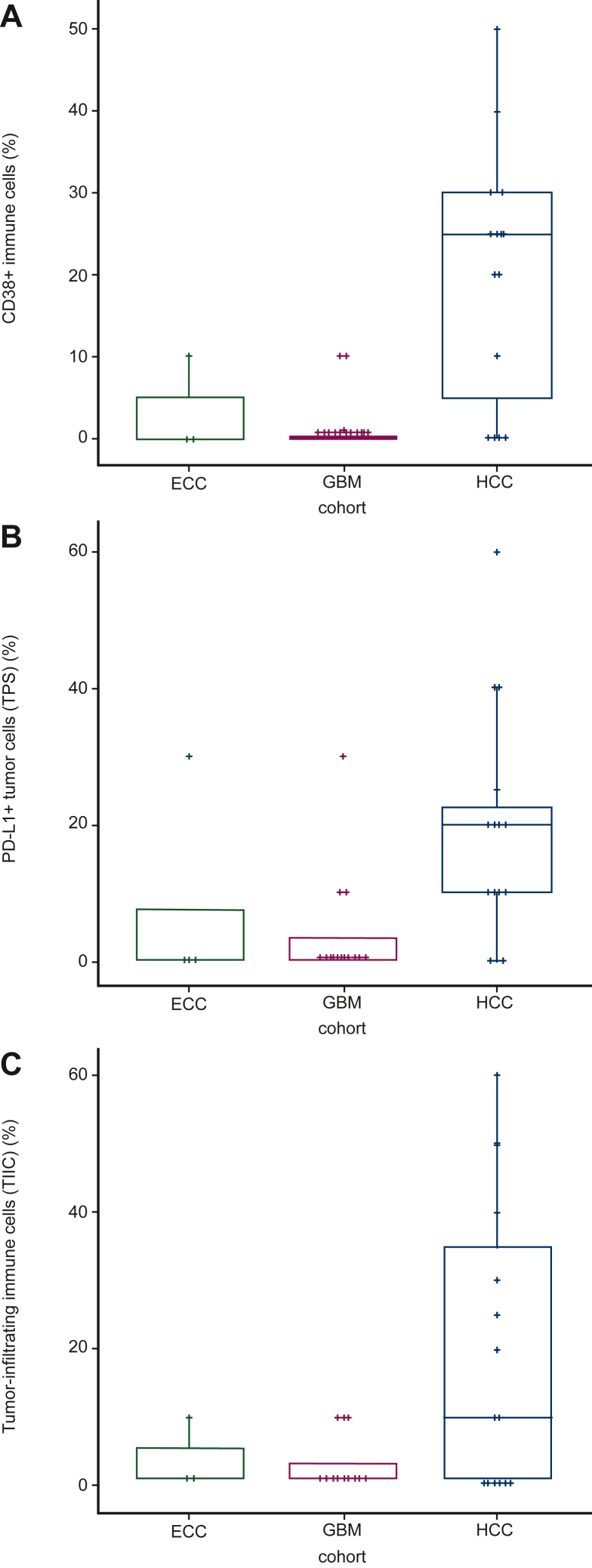
**Baseline levels of immune cells in patients with epithelial ovarian cancer (EOC), glioblastoma (GBM), and hepatocellular carcinoma (HCC).** Baseline levels of (A) CD38+ immune cells, (B) programmed death-ligand 1 (PD-L1) tumor positive score (TPS), and (C) tumor-infiltrating immune cells in the tumor microenvironment of patients with EOC, GBM, and HCC.

**Figure 2:**
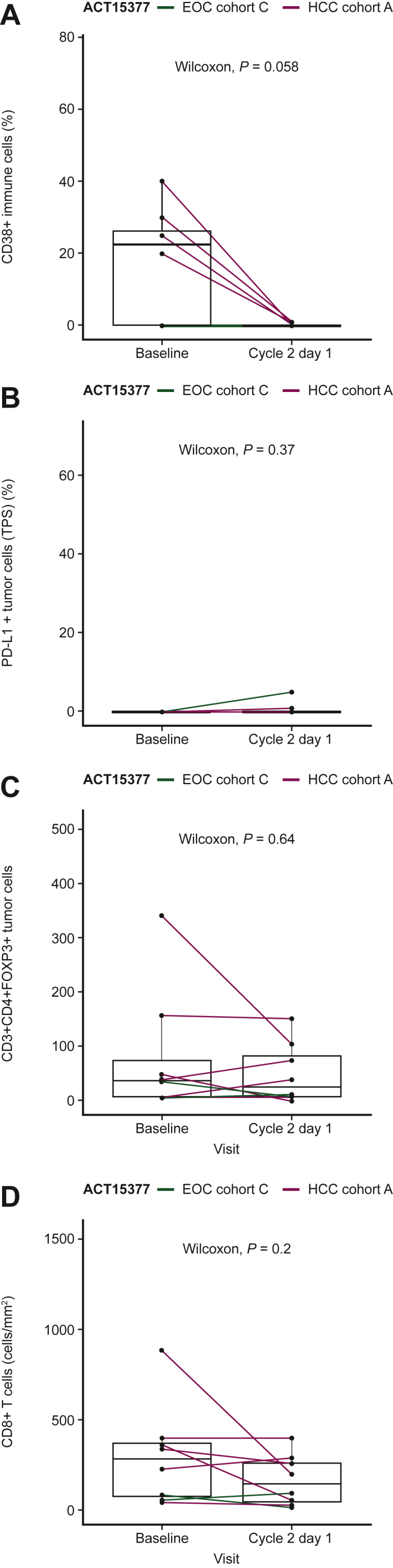
**Biomarker analysis of samples from patients with epithelial ovarian cancer (EOC) and hepatocellular carcinoma (HCC).** The isatuximab plus atezolizumab combination induces (A) a reduction of CD38+ immune cells; (B) non-significant modulation of programmed death-ligand 1 (PD-L1) expression on tumor cells [tumor proportion score (TPS)]; (C) non-significant modulation of regulatory T cells; and (D) non-significant modulation of CD8+ T cells in the tumor microenvironment of patients with EOC and HCC. ‘*P*’ stands for *P* value based on the Wilcoxon test.

Baseline tumor tissue from patients with EOC (pre-treatment screening biopsies) and GBM (archival) had low infiltration by immune cells, generally not expressing CD38 and PD-L1 (Figure 1). In contrast, HCC baseline samples showed a wide range of tumor-infiltrating immune cells (0%-60% of total cells present). Among HCC cases, detection of PD-L1-positive or CD38-positive infiltrating immune cells ranged from 0% to 50% and from 0% to 60%, respectively (Figure 1). No clinical signal was observed in patients with SCCHN; therefore, it was decided not to analyze biopsy samples. Pharmacodynamic effects were analyzed in the immune infiltrates from HCC and EOC cases where baseline and on-treatment tumor samples were available. Biopsies collected from patients with EOC and HCC revealed that tumor-infiltrating CD38+ immune cells were reduced and almost cleared by treatment [median (range), 22.5 (0-40) at baseline and 0 (0-1) at cycle 2 day 1; *n* = 8 (Figure 2)]. Isa + Atezo did not lead to significant modulation of PD-L1 expression in tumors or regulatory T cells in the TME (Figure 2).

### PK analysis

Isatuximab PK parameters from 95 patients (*n* = 16, EOC; *n* = 28, GBM; *n* = 25, HCC; *n* = 26, SCCHN) were consistent after the first administration across cohorts (Supplementary Table S1, available at https://doi.org/10.1016/j.esmoop.2022.100560). The mean isatuximab maximum observed concentration (C_max_) and area under the concentration versus time curve over the first 1-week dosing interval (AUC_0-168 h_) was 240 μg/ml and 22 600 μg·h/ml, respectively, with low variability (percent coefficient of variation for C_max_ and AUC_0-168 h_: 26% and 27%, respectively; Supplementary Figure S1, available at https://doi.org/10.1016/j.esmoop.2022.100560). A twofold increase in exposure [C_trough_ (concentration observed just before dosing)] was observed at the end of the weekly administration compared with the first administration. The exposure remained within the same magnitude during the every-3-week administration.

## Discussion

The present phase I/II study was designed to determine safety, tolerability, and preliminary efficacy of the anti-CD38 monoclonal antibody isatuximab given in combination with the anti-PD-L1 monoclonal antibody atezolizumab in advanced solid tumors. The aim of the study was to explore whether CD38 blockade may reshape the TME and enhance the activity of anti-PD-L1 therapy.

Cancer types selected for this trial included HCC and SCCHN, which are moderately immune-responsive to anti-PD-1/PD-L1 monotherapy, and GBM and EOC, which are immune-resistant cancers for which PD-1/PD-L1 axis blockade alone has not been shown to be effective.

The combination of Isa + Atezo was well tolerated, showing a manageable safety profile in line with that previously established for each agent as monotherapy. Importantly, no new safety signals were reported in these cohorts of immunotherapy-naive pre-treated patients with EOC, GBM, HCC, and SCCHN. No DLTs occurred and the RP2D was confirmed as isatuximab 10 mg/kg i.v. weekly for 3 weeks followed by every 3 weeks plus atezolizumab 1200 mg i.v. every 3 weeks. TRAEs were mostly mild to moderate in severity, manageable with supportive care, and reversible. The most common TEAEs were infusion reactions.

Previous studies have demonstrated the acceptable safety, tolerability, and antitumor activity of isatuximab as monotherapy, and in combination with pomalidomide or carfilzomib and dexamethasone in patients with MM.^8^, ^9^, ^10^ The safety and antitumor efficacy of atezolizumab has been confirmed in patients with solid tumors, including as monotherapy in patients with NSCLC, GBM, or SCCHN,^11^, ^12^, ^13^ and in combination regimens in patients with HCC.^14^^,^^15^

In the current small cohorts of pre-treated, immunotherapy-naive patients with EOC, GBM, HCC, or SCCHN, the combination of Isa + Atezo did not show a sufficient level of antitumor activity to expand enrollment and the study was stopped early as per protocol. This result seems to indicate that CD38-mediated immunosuppression may not be a relevant mechanism of primary resistance to PD-1/PD-L1 blockade, at least in these cancer types. The ORR and DOR observed in the HCC cohort of the current study are lower than those previously reported with the anti-PD-1 monoclonal antibody nivolumab given as monotherapy to patients who previously received sorafenib (12%; 9.9 months),^16^ showing no synergistic activity of PD-L1/CD38 combination blockade. With an ORR of 14%, activity of Isa + Atezo in the SCCHN cohort is similar to that previously seen with PD-1 blockade alone (ORR 13%-18%).^17^ Based on previous experiences with immune checkpoint therapy, no activity signals have been observed in GBM (ORR 0%) or EOC (ORR ∼5%).

Clinical pharmacodynamic evaluation revealed efficient target engagement of isatuximab by demonstrating treatment-mediated reduction of CD38+ immune cells in the TME. However, based on the present clinical data, CD38 inhibition does not seem to influence primary response to PD-L1 blockade in these patients. Although no new safety signals were observed, efficacy did not fulfill criteria to expand enrollment despite the evidence of target engagement of isatuximab.

PK exposure to isatuximab when given in combination with atezolizumab was comparable across solid tumor types. Similar results were observed when isatuximab was given in combination with cemiplimab (anti-PD-1) in patients with advanced NSCLC and metastatic castration-resistant prostate cancer,^18^ suggesting no effect of PD-1/PD-L1 blockade on isatuximab PK.

This study was limited by the small number of patients enrolled with each tumor type and the early study termination based on the interim analysis. Patients were not selected based on the presence of a putative target/biomarker, i.e. CD38 expression. The main strengths of the current study include the demonstration of acceptable safety and tolerability of Isa + Atezo, with biomarker analysis revealing clear target engagement of isatuximab. Additional studies are needed to further investigate underlying biomarkers that may inform treatment selection and predict benefit of combination therapy with anti-CD38 and anti-PD-1/PD-L1 agents in patients with solid tumors, including patients with progression after an initial response to PD-1/PD-L1 blockade.

In conclusion, despite the favorable safety profile, the combination of Isa + Atezo had limited activity in patients with the treatment-resistant or treatment-refractory solid tumors examined.
