# Supplementary material for: Isatuximab plus atezolizumab in patients with advanced solid tumors: results from a phase I/II, open-label, multicenter study
Source: ESMO Open. 2022 Aug 18;7(5):100562. doi: 10.1016/j.esmoop.2022.100562 (PMC9588873; doi:10.1016/j.esmoop.2022.100562)
Supplement: Supplementary Material [file mmc1.docx]

**Supplemental material**

**METHODS**

**Study assessments**

Any AEs specified below occurring during the first cycle of treatment, unless due to disease progression or an obviously unrelated cause will be considered a dose-limiting toxicity (DLT), if confirmed by the Study Committee. The duration of the DLT observation period will be longer for participants who delay initiation of Cycle 2 due to treatment-related AE for which the duration must be assessed in order to determine if the event is a DLT. The National Cancer Institute Common Terminology Criteria for Adverse Events version 4.03 will be used to assess the severity of AEs.

Hematological abnormalities are defined as any of the following:

- Grade 4 neutropenia for 7 or more consecutive days
- Grade 3 to 4 neutropenia complicated by fever (temperature ≥38.5°C on more than 1 occasion) or microbiologically or radiographically documented infection
- Grade 3 to 4 thrombocytopenia associated with clinically significant bleeding requiring clinical intervention

Non-hematological abnormalities

- Findings consistent with a Hy’s law case, consisting of all the following 3 components:
  - Grade ≥2 AST or ALT elevation simultaneous with Grade ≥2 total bilirubin elevation without initial findings of cholestasis (such as elevated serum alkaline phosphatase [ALP])
  - No other reason can be found to explain the combination of increased AST or ALT and bilirubin, such as viral hepatitis A, B, or C, pre-existing or acute liver disease (such as HCC or liver metastasis), or in absence of another drug capable of causing the observed injury
- Grade 4 non-hematologic AE, except:
  - Either Grade 4 AST, Grade 4 ALT or bilirubin elevation Grade 4 that improve to Grade ≤2 within 3 weeks of onset for participants with SCCHN, EOC, or GBM without liver metastasis
  - Either Grade 4 AST, Grade 4 ALT or bilirubin elevation Grade 4 for participants with liver metastasis or HCC with abnormal baseline value (ie, Grade 1 or 2)
- Grade 4 laboratory abnormality that is resolve within 3 days with or without therapeutic intervention.
- Grade 3 non-hematological AE lasting >3 days with optimal supportive care, except:
  - Grade 3 fatigue
  - Allergic reaction/hypersensitivity attributed to isatuximab or atezolizumab
  - Grade 3 laboratory abnormality that is asymptomatic
  - Either Grade 3 AST, Grade 3 ALT or bilirubin elevation Grade 3, for participants with liver metastasis or HCC with abnormal baseline value (ie, Grade 1 or 2)
  - Grade 3 nausea, vomiting, or diarrhea if well controlled by systemic medication within 7 days
  - Grade 3 immune-related AE that improves to Grade ≤1 within 3 weeks of onset
  - Grade 3 arthralgia that can be adequately managed with supportive care or that improves to Grade ≤2 within 7 days
  - Grade 3 elevation of serum creatinine that is not accompanied by elevations in blood urea nitrogen (BUN) or other signs of renal injury or that can be attributed to other etiologies such as disease-related obstructions
  - Grade 3 autoimmune thyroiditis or other endocrine abnormality that can be managed by endocrine therapy or hormone replacement
  - Grade 3 infusion reaction
  - Grade 3 skin rash that improves to Grade ≤1 within 7 days with appropriate supportive care.
- Delay in initiation of Cycle 2 more than 14 days due to treatment-related laboratory abnormalities/AE
- In addition, any other AE that the Study Committee deems to be dose limiting, regardless of its grade, may also be considered as DLT

**Statistical considerations**

The assumptions of response rate, the required sample sizes, and the number of responses at each stage are provided below for EOC, HCC, and SCCHN:

| **Indication** | **H0** | **H1** | **Sample size** | | **Number (%) of responses to**  **reject H0** | |
| --- | --- | --- | --- | --- | --- | --- |
|  |  |  | **Stage 1** | **Final** | **Stage 1** | **Final** |
| HCC and SCCHN | 15% | 30% | 26 | 59 | ≥5 (19.2) | ≥14 (23.7) |
| EOC | 20% | 40% | 17 | 37 | ≥4 (23.5) | ≥12 (32.4) |

EOC, epithelial ovarian cancer; H0, null hypothesis; H1, alternative hypothesis; HCC, hepatocellular carcinoma; SCCHN, squamous cell carcinoma of the head and neck.

The assumptions of progression-free survival at 6 months (PFS-6) and the required sample sizes at each stage are provided below for GBM:

| **Indication** | **H0** | **H1** | **Sample size** | | **Percentage of responses to**  **reject H0** | |
| --- | --- | --- | --- | --- | --- | --- |
|  |  |  | **Stage 1** | **Final** | **Stage 1** | **Final** |
| GBM | 20% | 35% | 31 | 65 | ≥19.4% | ≥27.7% |

GBM, glioblastoma; H0, null hypothesis; H1, alternative hypothesis.

**RESULTS**

**Online supplemental figure 1.** Isatuximab C_max_ (A) and AUC_0-168h_ (B) after the first administration of isatuximab at 10 µg/kg. AUC_0-168h_, area under the concentration versus time curve over the first 1-week dosing interval; C_max_, maximum observed concentration; EOC, epithelial ovarian cancer; GBM, glioblastoma; HCC, hepatocellular carcinoma; SCCHN, squamous cell carcinoma of the head and neck.


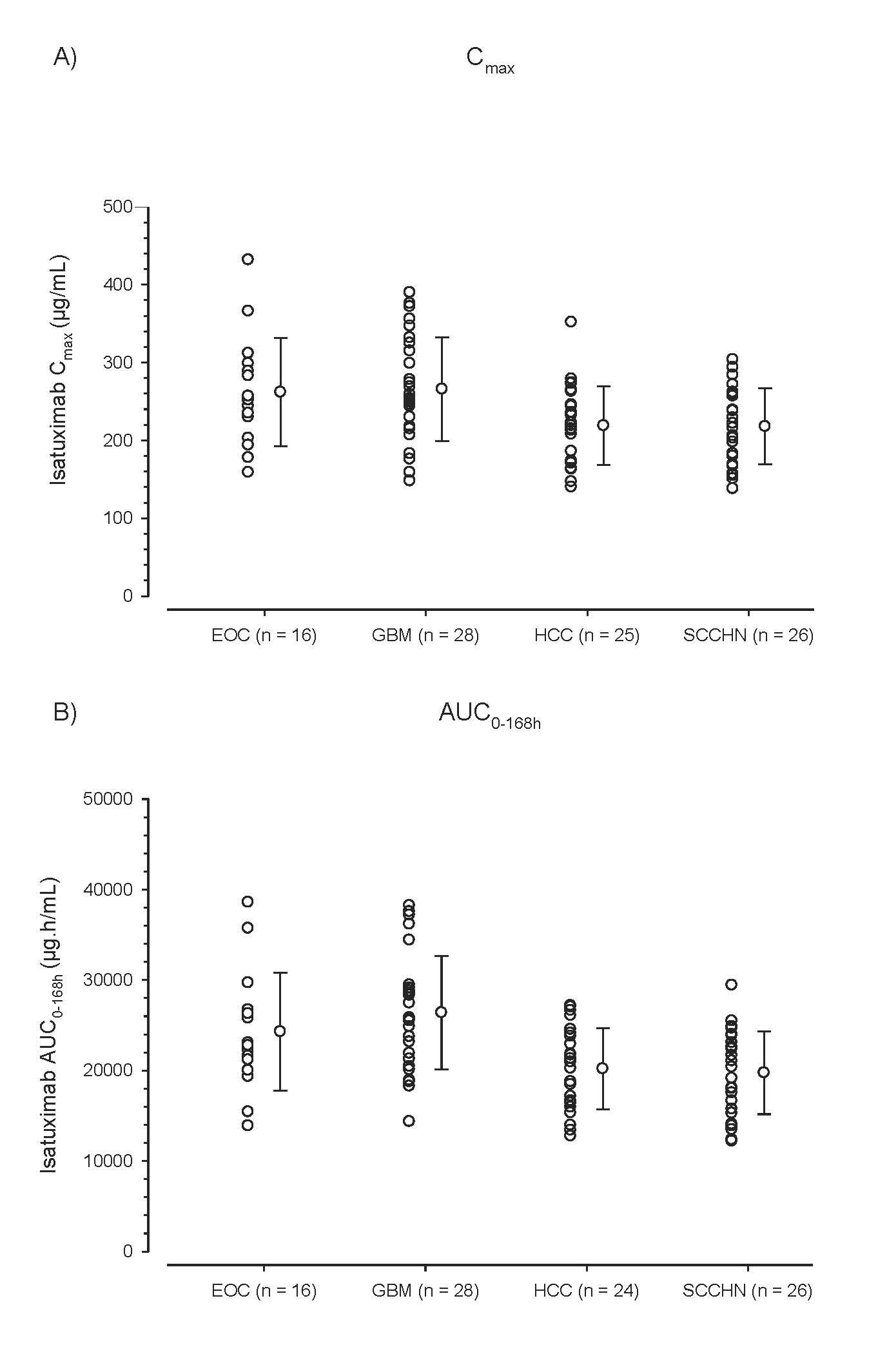


Online supplemental table 1. Isatuximab plasma PK parameters after the first administration of isatuximab at 10 µg/kg.

| Mean ± SD (geometric mean) [CV%] | Isatuximab PK parameters | | | | |
| --- | --- | --- | --- | --- | --- |
|  | **EOC** | **GBM** | **HCC** | **SCCHN** | **All cohorts** |
| N | 16 | 28 | 25 | 26 | 95 |
| C_max_  (μg/mL) | 262 ± 69.7 | 266 ± 66.7 | 219 ± 50.5 | 218 ± 48.7 | 240 ± 62.2 |
|  | (254) [27] | (258) [25] | (214) [23] | (213) [22] | (232) [26] |
| t_max_^a^  (h) | 6.83 | 4.13 | 4.00 | 3.37 | 4.54 |
|  | [3.00-8.83] | [2.65-8.38] | [2.42-7.92] | [2.08-8.95] | [2.08-8.95] |
| AUC_0-168h_  (μg•h/mL) | 24,300 ± 6500 | 26,400 ± 6270 | 20,200 ± 4510^b^ | 19,800 ± 4590 | 22,600 ± 6130^c^ |
|  | (23,500) [27] | (25,700) [24] | (19,700) [22] | (19,200) [23] | (21,800) [27] |

^a^Median (Min-Max) values.

^b^n=24.

^c^n=94.

AUC_0-168h_, area under the concentration versus time curve over the first 1-week dosing interval; C_max_, maximum concentration observed; CV%, percent coefficient of variation; EOC, epithelial ovarian cancer; GBM, glioblastoma; HCC, hepatocellular carcinoma; PK, pharmacokinetic; SCCHN, squamous cell carcinoma of the head and neck; SD, standard deviation; t_max_, time to reach maximum concentration.
